# Unexpected periosteal bone apposition including newly embedded osteocytes occurs around a mouse calvaria critical defect, independently of the presence of biomaterials

**DOI:** 10.1038/s41598-026-58356-7

**Published:** 2026-07-02

**Authors:** Mathilde Palmier, Marlène Maître, Hélène Doat, Thierry Lesté-Lasserre, Steve Peuble, Frederic Gallice, Mathilde Fénelon, Claudine Boiziau, Delphine B. Maurel

**Affiliations:** 1 Inserm, Univ. Bordeaux, BioTis Laboratory UMR 1026, Bordeaux, 33 000 France; 2 Inserm, Univ. Bordeaux, Neurocentre Magendie UMR 1215, Bordeaux, 33 000 France; 3 Mines Saint-Etienne, CNRS, Univ Jean Monnet, Univ Lumière Lyon 2, Univ Lyon 3 Jean Moulin, ENS Lyon, ENTPE, ENSA Lyon, UMR 5600 EVS, F-42023 Saint-Etienne, France

**Keywords:** Calcium-phosphate biomaterials, Xenograft, Bone regeneration, ICP-AES, Biotechnology, Cell biology, Materials science, Medical research

## Abstract

Osteocytes embedded in mineralized bone have their network disrupted when a bone injury occurs. However, their role in regeneration is still unclear. Bone substitutes, including bioceramics and bone-derived extracts, are commonly implanted in critical-sized defects due to their bioactive properties that promote healing, yet few studies have examined their effects on osteocytes in vivo. We studied early repair phases of a critical defect model in adult male mice calvaria, with or without biomaterial implantation (β-TCP, bovine bone). Using microCT we determined that, after 14 days, bone formation had mainly occurred along the surface of existing bone, increasing its thickness by a factor of 1.6, independently of the biomaterial presence. Using HE staining and fluorescence imaging, we described the newly formed bone and showed the presence of recently embedded osteocytes. We specifically collected osteocytes close, and distant from the defect, using laser-assisted microdissection and analyzed their gene expression. We show that *IL6* was mainly dependent on the delay after surgery whereas *Dmp1* was spatially regulated. Thus, even with limited bone formation in the defect, bone apposition occurs on the inner and outer periosteal surfaces of the calvaria, a phenomenon that may have been overlooked in the development of bone repair strategies.

**Supplementary Information:**

The online version contains supplementary material available at https://doi.org/10.1038/s41598-026-58356-7.

## Introduction

While bone tissue has an inherent ability to heal, complications such as delayed union or non-union still affect many patients and impair their quality of life^1^. To support the healing process, the gold standard is to proceed to an autograft: bone is collected from a healthy site in the patient and used to fill the defect. Despite its efficacy, the restricted quantity of bone available and the risk of comorbidity at the donor site limit the applications. Allografts (obtained from human donors) and xenografts (primarily derived from bovine or porcine tissues) can also be used, although their healing performances are lower compared to autografts. Alternative options such as bone substitutes, are currently commercially available, but none can fully replace the gold standard, which emphasizes a need for further improvements^2,3^.

Therefore, understanding the bone healing process and the key cellular and molecular players involved is essential. Bone healing occurs through several overlapping phases driven by the coordinated activity of different cell types responding to specific molecular signals. During the inflammatory phase, cytokines such as interleukin-1 (IL-1), interleukin-6 (IL-6), and tumor necrosis factor-α (TNF-α) are released by immune cells, promoting the recruitment of inflammatory cells and mesenchymal stem cells, as well as stimulating angiogenesis. Within a few days, the bone formation phase begins, primarily originating from the defect edges and proceeding via two mechanisms: endochondral and intramembranous ossification, the latter predominating in the calvaria. This process leads to the formation of immature, porous bone within a few weeks^4^. Subsequently, the remodeling phase transforms this immature tissue into mature, lamellar, and functional bone. Bone remodeling is orchestrated by osteocytes, which regulate osteoblast and osteoclast activity through their dendritic networks and secreted factors, as reviewed by Dallas et al.^5^. Thus, in the months following injury, osteocytes contribute to the completion of the healing process. Emerging evidence also suggests that osteocytes may play a role in the early phases of regeneration via IL-6, DMP1, and sclerostin^6–8^. However, their function remains unclear, particularly in the context of critical-size defects in the calvaria^9^.

Calcium-Phosphate (CaP) biomaterials are widely used as bone substitutes for research and in clinics. Their chemical composition is close to the bone mineral, they are natural or synthetic and have modifiable shapes and sizes. Their ability to dissolve and release Ca^2+^ and PO_4_^3−^ ions is particularly interesting because it gives them bioactive properties^10^. Calcium and phosphate ions have been shown to promote cell differentiation, migration and to regulate activity in mesenchymal stem cells, osteoblasts, and osteoclasts^10,11^. Their effects on osteocytes have not been extensively studied, despite the fact that osteocytes respond to changes in extracellular calcium concentration and possess mechanoresponsive calcium channels that regulate bone formation. Ishihara et al. demonstrated that extracellular calcium levels influence osteocyte cell–cell communication via Connexin 43 (Cx43) gap junctions^12^, and *Cx43* expression in both osteoblasts and osteocytes is required for bone repair in mice^13^. Additionally, osteocytes have been shown to mediate bone formation through the mechanosensitive calcium channel PIEZO^14^. In their study, Zhang et al. even reported that TriCalcium Phosphate particles could promote osteocyte death in the calvaria^15^. Therefore, understanding how osteocytes contribute to bone regeneration could guide the development and optimization of more effective bone substitutes.

In this study, we created a critical-size defect model in the calvaria of adult male mice, filled with or without CaP biomaterial, and assessed the early and intermediate repair phases. We used HE staining and microCT to quantify bone formation at different sites of the calvaria. To study the osteocytes, we used fluorescence imaging, as well as a laser-assisted microdissection technique^16^ to collect them specifically and subsequently analyze their mRNA expression in the different conditions.

## Results

### In vitro ion release profiles from the β−TCP and xenograft biomaterials

We chose to study the effect of two biomaterials provided as granules (250 μm to 1 mm) by the manufacturer (SEM images in supplementary figure S1). We studied a xenograft (“Xeno”) made of natural bovine bone, highlighted by the presence of osteocyte lacunae, and a synthetic β−TCP made of powder grains forming microporosities after sintering.

Since these biomaterials are known to release calcium and phosphate ions, we measured the concentration of calcium and phosphorus elements released after 72 h of immersion into ultrapure water (Table 1). β−TCP mainly released calcium, whereas the xenograft released calcium and phosphorus. Interestingly, the concentration of calcium released by β−TCP was 12 times higher than for the xenograft. We concluded that, in vivo, the β-TCP granules might release a higher amount of calcium ions but a lower amount of phosphate than Xeno granules.

**Table 1:**
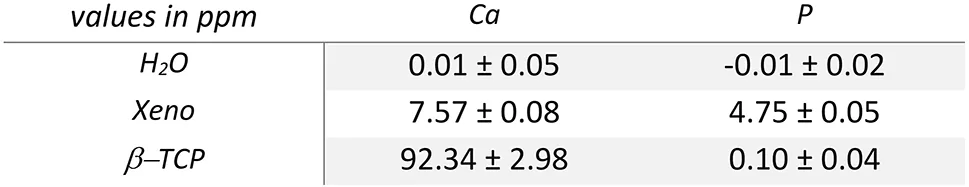
Analysis of in vitro calcium (Ca) and phosphorus (P) release. Results of ICP-AES analysis in ultrapure water.

### Bone formation occurred mainly around the defect after 14 days

**Fig. 1:**
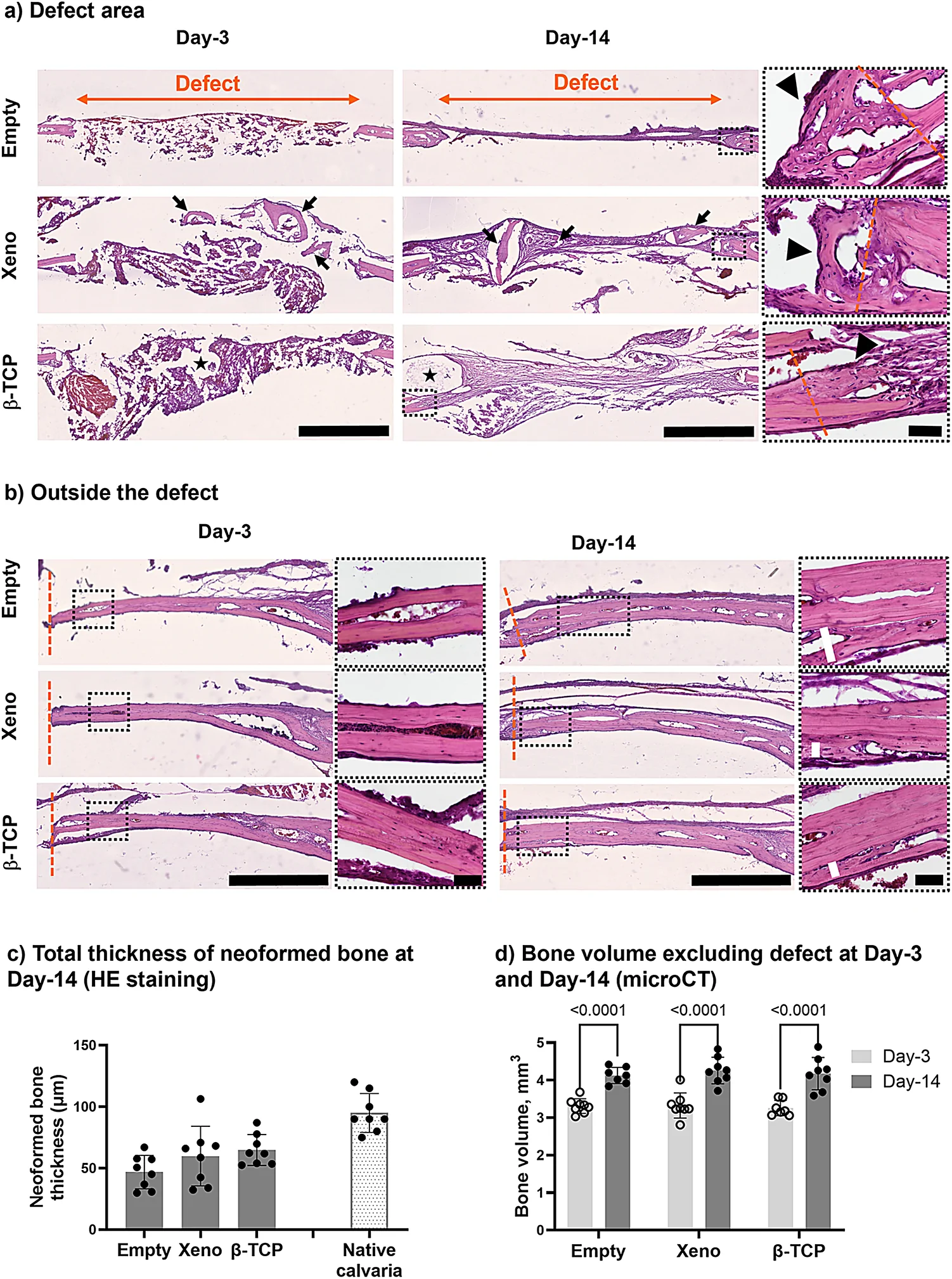
Hematoxylin-Eosin (HE) staining on sagittal sections and microCT analysis for the three conditions (Empty, Xeno, β-TCP). **(a)** Calvaria defects on Day-3 and Day-14; insets on the right show magnification of the defect edge at Day-14. Black arrows: Xeno biomaterial; black stars: locations of β-TCP granules; black arrow heads: newly formed bone at the edge of the defect; orange dashed line: limits of the initial defect. **(b)** Calvaria images around the defect on Day-3 and Day-14; white bars highlight newly formed bone on Day-14. **(c)** Quantification of the thickness of newly formed bone along the calvaria on Day-14. The mean thickness of native calvaria (95 ± 15 μm) is shown on the right for comparison. **(d)** Quantification after microCT imaging of the new bone volume formed, excluding the defect area and granules, on Day-3 and Day-14. The data are presented as mean ± SD, *n* = 8 in each group, statistical analyses: Two-way ANOVA and Kruskal-Wallis. Scale bars: 1 mm, 50 μm (insets).

The Xeno and β-TCP granules were implanted at the site of the critical bone defect; then the calvaria samples were analyzed 3 and 14 days after surgery (“Day-3” and “Day-14”) and compared to the defect left empty. On Day-3 and Day-14, no bone formation was visible around the bovine bone granules (Fig. 1a, black arrows) and β-TCP (Fig. 1a, black stars), and very few bone formation was visible at the edge, only on Day-14 as shown on Fig. 1a (black arrow heads).

However, on Day-14, we identified new bone apposition on the native lamellar bone around the defect (Fig. 1b, white bars). The thickness of this bone formed after 14 days was measured on sagittal sections stained with Hematoxylin-Eosin (Fig. 1c). The mean thicknesses along the calvaria were 47 ± 13 μm (Empty), 60 ± 24 μm (Xeno), and 65 ± 13 μm (β-TCP), without significant differences between the three conditions. This bone neoformation was substantial, representing 50 to 68% of the native calvaria thickness (95 ± 15 μm, Fig. 1c). In addition, bone formation around the defect was quantified using microCT, segmenting and excluding the defect volume and the biomaterial granules for the different conditions (Empty, Xeno and β-TCP). We thus confirmed that an additional bone volume was present on Day-14 compared to Day-3 and it was similar in all the conditions (Fig. 1d, supplementary figure S2).

## Site-specific periosteal bone apposition

The analysis of the mean thickness of the periosteal bone apposition along the calvaria 14 days after surgery revealed that it was site-specific.

**Fig. 2:**
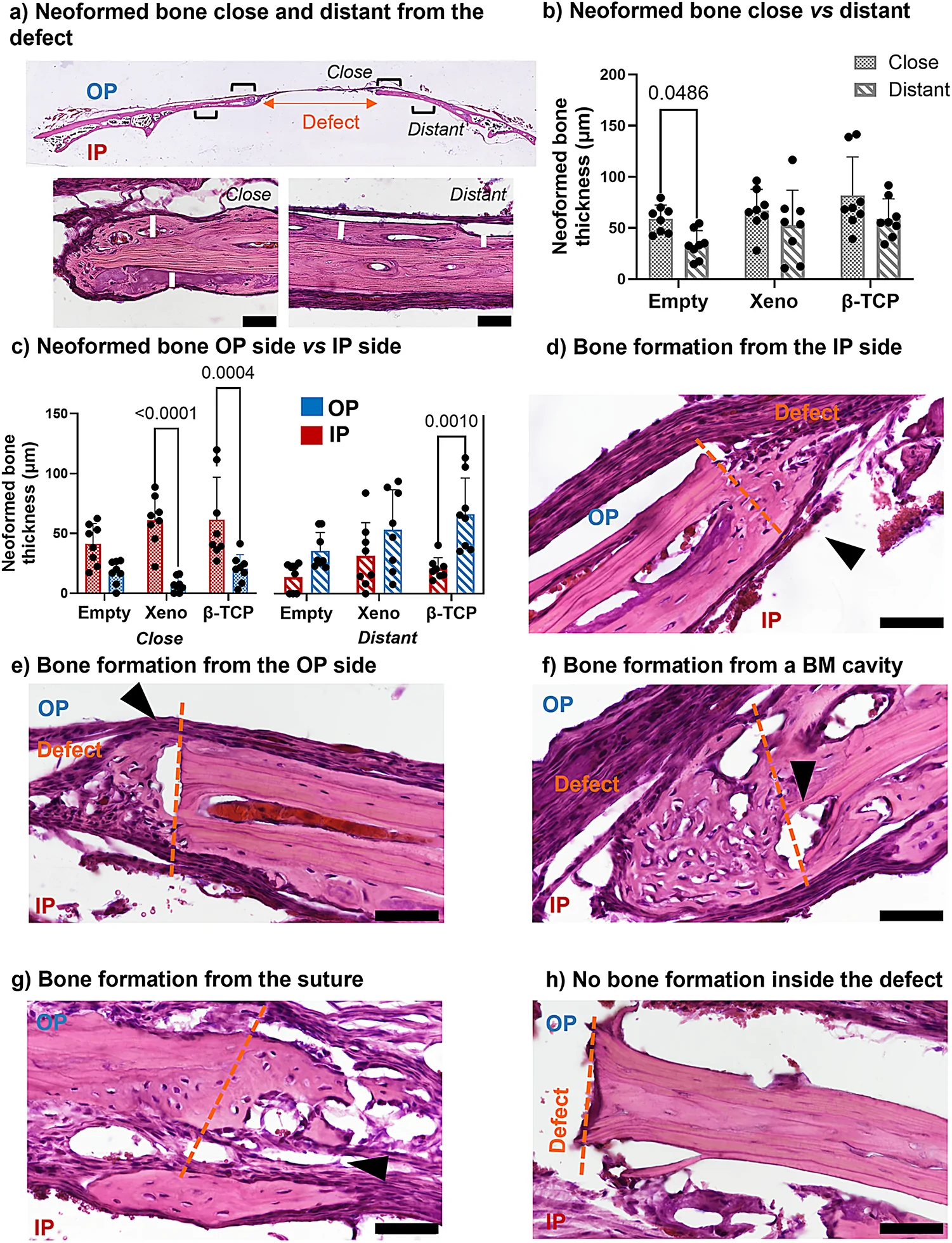
Periosteal bone apposition on Day-14 in the three conditions (Empty, Xeno, β-TCP).** (a)** Phase contrast imaging of HE staining (sagittal sections, Empty condition), white bars indicate newly formed bone on the outer periosteum (OP) and inner periosteum (IP) sides, close and distant from the defect. **(b)** Comparison of the thickness of newly formed bone close (0–600 μm) and distant from the defect (1.0–1.6 mm). **(c)** Comparison of the thickness of newly formed bone on the OP and IP sides, close and distant from the defect. The data are presented as mean ± SD; *n* = 8 in each group, statistical analyses: Two-way ANOVA. Bone formation inside the defect might be initiated from **(d)** the IP side, from **(e)** the OP side, from **(f)** bone marrow cavities (BM), or from **(g)** the suture. **(h)** No bone formation was also observed inside some defects. Scale bars: 50 μm.

In the Empty condition, as seen in Fig. 2a and b, the mean thickness of the new bone close to the defect (within 600 μm from the defect edge) was higher than the mean thickness distant from the defect (1.0–1.6 mm from the defect edge). In the Xeno and β-TCP conditions, no significant differences were observed (Fig. 2b).

We also analyzed separately the mean thickness of the new bone formed on the outer periosteum (OP), and on the inner periosteum (IP) (Fig. 2a). We found that the bone formation close to the defect was higher on the IP side than on the OP side in the Xeno and β-TCP conditions (Fig. 2c). The bone formation distant from the defect was significantly higher on the OP side than on the IP side in the β-TCP condition only (Fig. 2c).

Then, when present, we determined the origin of bone regeneration inside the defect (Fig. 2d-g). In 85% of the cases, the bone apposition on the IP side extended inside the defect (Fig. 2d), although the OP (Fig. 2e), bone marrow (BM) cavities cut by the trephine (Fig. 2f) or suture (Fig. 2g) could also initiate some bone formation. In rare cases, although bone apposition occurred around the defect on the IP side, it did not give rise to bone formation inside the defect as shown in Fig. 2h. Nevertheless, bone formation on the IP side below 250 μm from the defect edge was identified in all animals (regardless of the condition, Empty, Xeno or β -TCP). In contrast, on the OP side in the same area surrounding the defect, only 60% of the “Empty” and “β-TCP” samples and 25% of the “Xeno” samples exhibited bone apposition, which explains that newly formed bone inside the defect was predominantly detected as originating from the IP side of the calvaria.

## Osteocyte populations on Day-3 and Day-14

**Fig. 3:**
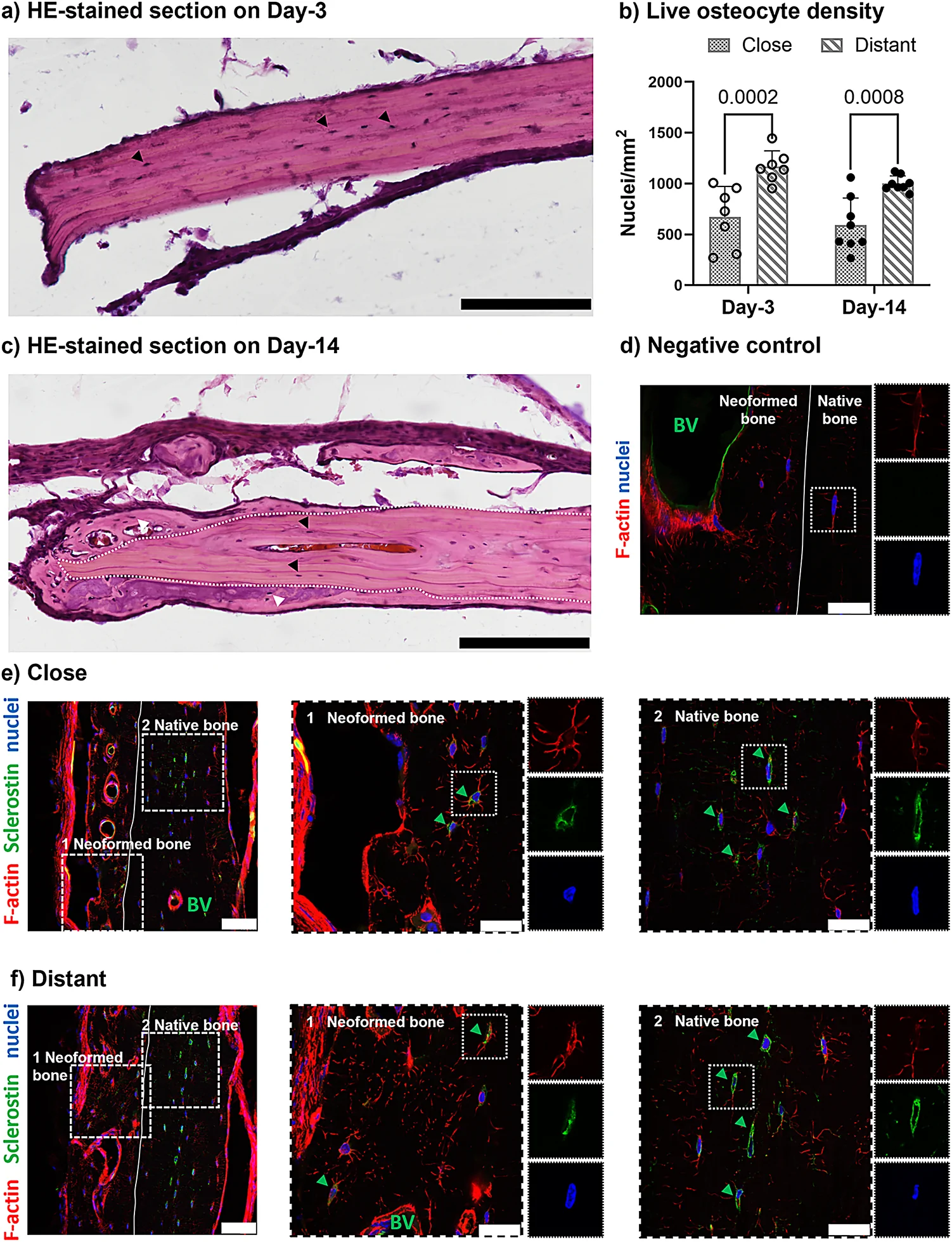
Osteocyte populations on Day-3 and Day-14. **(a)** HE-stained section showing embedded cell nuclei on Day-3 (black arrow heads). **(b)** Quantification of the number of osteocyte nuclei per mm^2^ in the native bone on Day-3, and Day-14, close (0–600 μm) and distant from the defect (1.0–1.6 mm). The data are presented as mean ± SD, *n* = 7/8, statistical analyses: Two-way ANOVA. **(c)** HE-stained section on Day-14 showing osteocytes in native bone (black arrow heads) and cells in neoformed bone (white arrow heads). The native bone limit is shown with the white dashed line. **d-f)** Phalloidin (red) and sclerostin staining (green and green arrowheads) of areas in native and neoformed bone **e)** close to the defect, **f)** distant from the defect, and **(d)** shows the negative control of sclerostin immunolabeling. Blood vessels (BV) are green due to GFP expression in endothelial cells in the Flk1-GFP mice. Scale bars: 100 μm (a, c), 50 μm (e, f, left), and 20 μm (d, e-f insets).

Using HE staining and counting nuclei of cells present in lacunae (Fig. 3a-c black arrow heads), we quantified osteocytes in the native bone three days post-surgery and found a lower density of osteocytes close to the defect compared to distant from the defect (0–600 μm vs. 1.0–1.6 mm), in the Empty condition. Osteocyte densities on Day-3 and Day-14 were similar (Fig. 3b).

We then focused on osteocytes in the newly formed bone on Day-14 (Fig. 3c, white arrow heads). The embedded cells close and distant from the defect (Fig. 3d-f) presented numerous dendrites highlighted with the red phalloidin staining and were positive for sclerostin immunolabelling (green), allowing to identify them as osteocytes. Note that the Flk1-GFP mice express GFP in endothelial cells, explaining the green labeling in blood vessels (Fig. 3d, negative control of the sclerostin immunolabeling).

## Osteocyte gene expression on Day-3 and Day-14

**Fig. 4:**
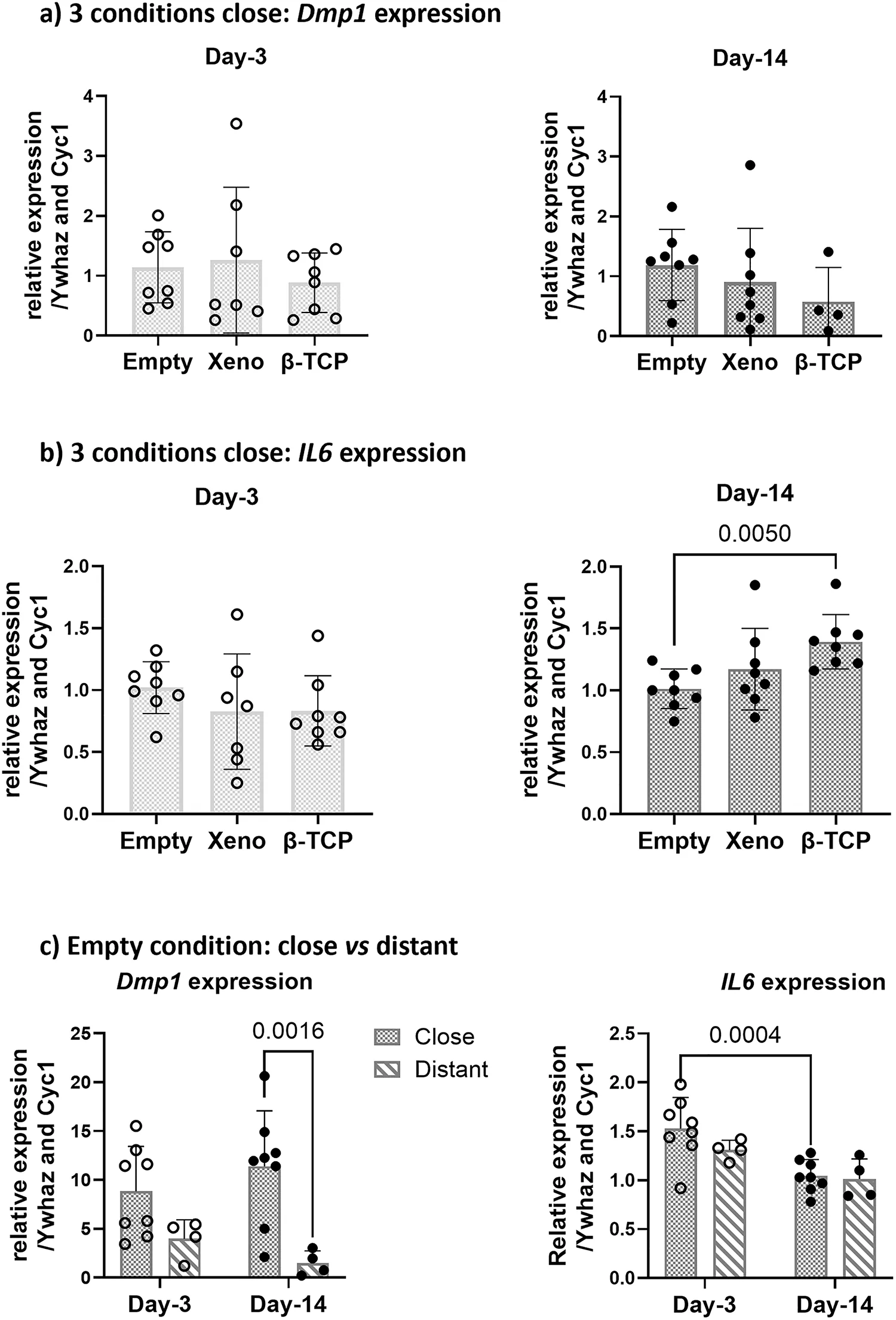
Analysis of *Dmp1* and *IL6* gene expression in osteocytes, 3 and 14 days post-surgery.**a-b)** Osteocytes were collected close to the defect for the Empty, Xeno and β-TCP conditions.**c)** Osteocytes were collected close and distant 600 μm from the empty defect, until the next suture. The data are presented as mean ± SD,*n* = 8 in each group except*n* = 4 in the “distant” groups, statistical analyses: Kruskal-Wallis and Two-way ANOVA. Depending on the comparison, different groups were used as reference (see Materials and methods).

We collected by laser-assisted microdissection the osteocytes from the native and the newly formed bone, in the area within 600 μm from the defect edge (close) and measured their gene expressions 3- and 14-days post-surgery. We analyzed six different genes (supplementary Table 1) in all the conditions (Empty, Xeno and β-TCP): four genes were chosen as expressed by osteocytes (*Sost*,* Phex*,* Dmp1*, and *E11*) and marker of their differentiation stage^5^, *Vegfa* as an angiogenic growth factor, and the *IL6* cytokine as an inflammatory marker in addition to its role in bone remodeling, both also expressed by osteocytes^17,18^. Laser microdissection on bone is challenging, even more in calvaria and in very specific areas, which limits the number of osteocytes collected per hour of dissection. Out of the six genes studied, only two gave reliable and reproducible results: *Dmp1* and *IL6*. We found that the expression of *Dmp1* was similar between the three conditions, on Day-3 and Day-14 days (Fig. 4a). The expression of *IL6* was similar between the conditions on Day-3, and it was slightly but significantly up-regulated in the β-TCP condition compared to the Empty one on Day-14 (Fig. 4b).

We also assessed whether the osteocyte distance from the defect edge had an impact on their gene expression 3 and 14 days post-surgery. Thus, additionally to the osteocytes collected within 600 μm close to the defect, we collected osteocytes further than 600 μm and until the next suture in the Empty condition. We found that *Dmp1* expression was up-regulated close to the defect compared to further away, 14 days post-surgery (Fig. 4c). Regarding *IL6*, its expression was slightly but significantly up-regulated close to the defect, 3 days after the surgery compared to 14 days post-surgery (Fig. 4c).

## Discussion

By studying the early repair phases of a calvaria critical defect, we reported that surprisingly, after 14 days, bone formation had mainly occurred on the surface of the native bone. In this newly formed bone, we showed the presence of recently embedded osteocytes adding to the populations originally present in the calvaria. In the native bone, the osteocyte density three days after surgery was lower close to the defect (within 600 μm) than distant from the defect (1 to 1.6 mm from the defect edges). This aligns with findings by Liu et al., who reported a lower percentage of lacunae including osteocytes close to the fracture than at distant sites in a murine rib fracture model^6^. Additionally, we used laser-assisted microdissection to collect osteocytes close and distant from the defect, without any contamination by bone marrow cells or bone lining cells. The subsequent RT-qPCR enabled us to analyze *Dmp1* and *IL6* expressions, which confirmed the impact of the defect creation on osteocytes. When combining these analyses with the implantation of CaP ion releasing biomaterials (β-TCP, or bovine bone granules), we did not evidence any major effect neither on the periosteal bone apposition nor on the osteocyte *Dmp1*, and *IL6* expressions.

We chose to study early timepoints to match the early and intermediate healing phases described for mice: 3 days post-surgery, corresponds to the inflammatory stage and 14 days post-surgery corresponds to the early stage of bone formation^19^. When analyzing bone formation within the defect, the IP was the main origin of the neoformed bone (Fig. 2e-g). This is consistent with the work of Bixel et al^20^. who showed that osteoprogenitors originated from the IP side of the calvaria in a non-critical bone defect in mice, with vascularization preceding progenitor migration, and matrix mineralization. Recently, Zhai et al. also showed that the bone formation of a cranial defect in mice was three times larger on the IP than on the OP surface, linked to the higher vessel density on the IP side^21^. However, we also observed bone neoformation from regions containing osteoprogenitors: the OP, bone marrow cavities and suture when close to the defect^22–26^. This is consistent with prior studies showing that sutures, periosteum and bone marrow provide a reservoir of mesenchymal stem cells^25,27^.

Contrasting with the defect site, the surface of the calvaria exhibited highly effective bone neoformation. Indeed, the area surrounding the defect thickened by a factor of 1.6, with two-thirds produced on the IP side. Interestingly, we estimated that within the 1.1 mm distance surrounding the 3.5 mm diameter defect, the same volume of bone was produced as would be required for a complete calvaria repair (supplementary figure S3). Bone apposition on the OP side was also described by Saulacic et al.^28^ on rat calvaria after periosteal elevation by an implanted device gradually separating the OP from the bone. Control animals that were sham operated also displayed a thickened calvaria (on the OP side), albeit at a lower level. However, in their experiments, no bone formation was observed on the inner side, supporting the hypothesis that the bone apposition that we observed on the IP side was caused by the defect. The contribution of the periosteum to bone repair is well established, particularly in long bones, where it serves as a major source of osteochondral progenitors during fracture healing. Colnot demonstrated that periosteal injury is sufficient to trigger endochondral bone formation, underscoring the intrinsic regenerative potential of periosteal cells^29^. Consistent with this, Duchamp de Lageneste et al. showed that skeletal stem cells residing within the periosteum of murine long bones possess a higher regenerative capacity than bone marrow–derived stem cells, further highlighting the dominant role of the periosteum in skeletal repair^30^. Together, these observations support the notion that periosteal activation contributes to bone formation on the OP side.

We confirmed that the new bone contained cells with an osteocytic morphology and expressing sclerostin (Fig. 3). Thus, the defect created a disruption in the osteocyte network, resulting in distinct osteocyte populations: osteocytes in the native bone in direct proximity to the defect and therefore most exposed to the structural discontinuity, osteocytes in the native bone located farther away and therefore less directly exposed, and newly embedded osteocytes that arose between Day-4 and Day-14. Different osteocyte populations close and farther from the bone damage have been described in the ribs and ulnae of rodents as having different protein profiles using immunohistochemistry^6,31^. mRNA analysis from snap-frozen bone with enriched osteocyte population was mainly used to describe their gene expressions at different time points^7,8,31^. In our study, we specifically isolated osteocytes using laser-assisted microdissection either from the area close to the defect or distant from the defect and were able to analyze the expression of *IL6* and *Dmp1*.

Regarding *Dmp1*, we found a similar expression close and distant from the defect at Day-3, but it was up-regulated close to the defect compared to distant at Day-14. Possibly, the osteocytes embedded in the newly formed bone strongly expressed *Dmp1* at Day-14, and the greater bone neoformation in the vicinity of the defect on both periosteal surfaces (59 ± 13 μm close *versus* 33 ± 14 μm distant) might explain part of the difference between close to and distant from the defect. In the rib, Liu et al. showed that the number of DMP1-positive osteocytes was similar close and distant from the defect 2, 4, and 7 days post-lesion and did not quantify at later time points^6^. When comparing to a model in long bone, the expression of *Dmp1* was shown to be up-regulated 7 and 14 days compared to day 0 after surgery in a non-critical mid-diaphyseal defect in the mouse tibia^7^.

Regarding *IL6* expression, we found that it was slightly higher on Day-3 than on Day-14 (factor of 1.5, Fig. 4c). This suggests that despite a significant difference between Day-3 and Day-14, calvaria osteocytes show a rather low increase of *IL6* expression in response to the critical defect created in our model. In contrast, Caetano-Lopes et al.^32^ reported a substantial increase in *IL6* expression from pulverized human trabecular hip bone pieces at the fracture site between the first 3 days post-fracture compared with 8 days after the bone injury. In a stress fracture model in the ulna, IL-6–positive cells were predominantly detected in the woven bone formed at the exit site of the fracture. Notably, the number of IL-6–positive cells was significantly lower at 7 days than at 4 days post-injury^8^. Although informative, comparisons between critical defect models and stress-induced injury models should be interpreted with caution. Mechanical loading alone can directly induce microdamage in osteocytes and alter their expression profiles, independent of bone damage. For example, apoptotic osteocytes have been reported in the vicinity of stress fractures, which may result from the defect itself, the applied loading, or a combination of both^8,31,33^.

This study was limited by the low amount of isolated mRNA after laser-microdissection leading to a low number of genes that could accurately be analyzed. In addition, due to this limitation, the neoformed bone was not analyzed separately from the native bone, preventing from understanding the contribution of each osteocyte population described. New technics such as spatial transcriptomics could be considered to overcome this limitation in the future^34^. Additionally, osteocyte morphology, lacunar alignment and transcriptional profiles differ between long bones and the calvaria^35,36^. Consequently, repair responses to injury may not be directly comparable, highlighting the need to also investigate critical-size defects in long bones.

In parallel, we chose to investigate the impact of calcium-phosphate releasing granules on osteocytes. In the case of the xenograft, the Ca/P ratio of the released ions was 1.24, which is lower than the Ca/P ratio of the solid-state reported as ranging from 1.33 to 2.31^37^. It indicates incongruent dissolution controlled by surface reactions and solution-solid equilibria^38,39^. Of note, Lee et al. reported a Ca/P ratio of 1.22 for a bovine-derived bone graft measured by ICP-MS; however, the dissolution protocol used prior to analysis was not described limiting the comparison^40^. For the β-TCP, calcium was predominantly detected in solution. This behavior is likely related to the sintering process, which is known to modify the surface chemistry by forming a calcium-rich layer that would dissolve first^41^. Despite the in vitro evidence of calcium and phosphate ion release from the implanted granules, no effect of their presence on osteocytes was detected.

**Fig. 5:**
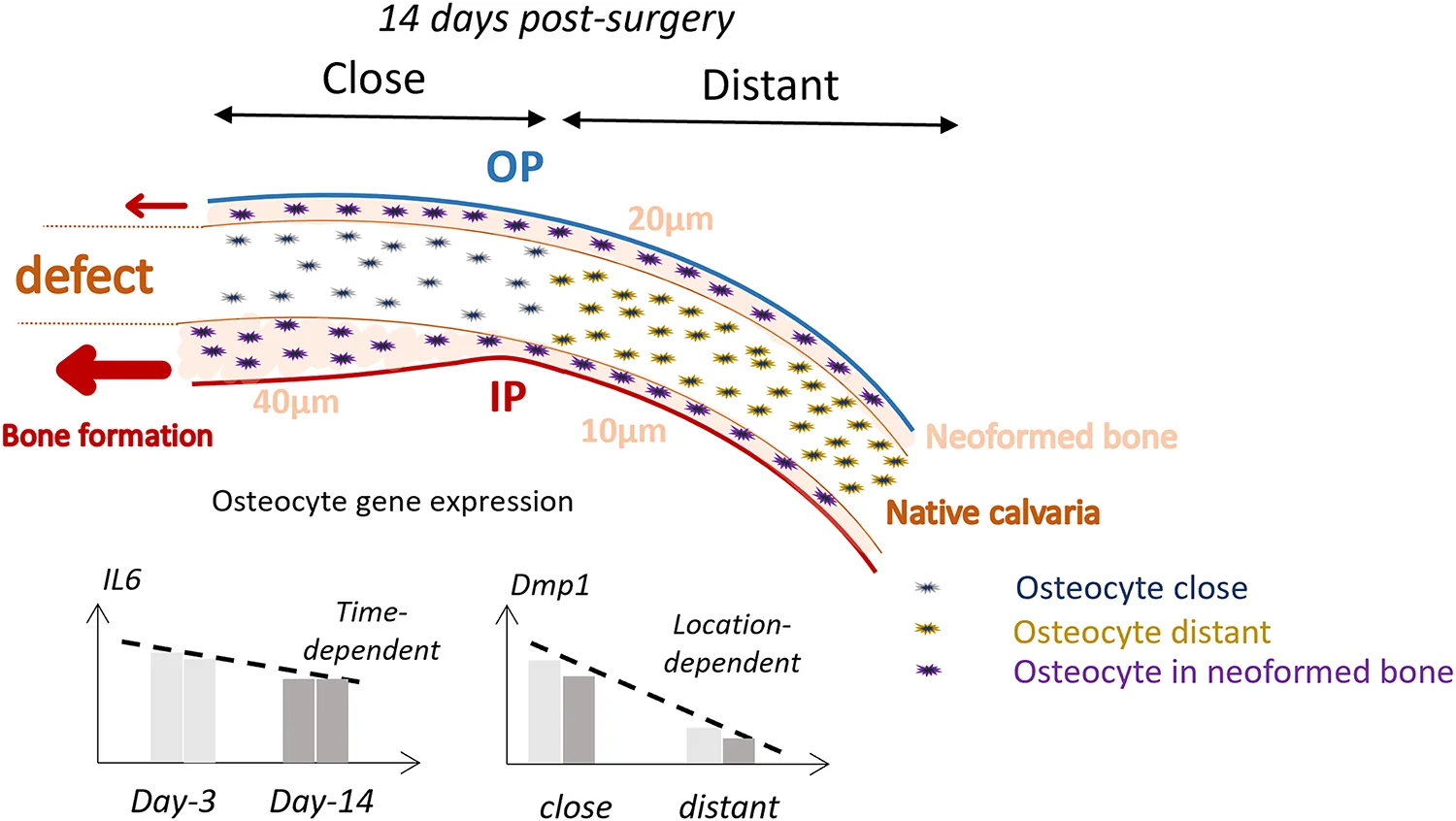
Schematic summary of the bone apposition on both periosteal sides of the calvaria in the Empty condition. The osteocytic expression of *IL6* and *Dmp1* is modulated temporally or spatially, respectively, during the early stages of bone neoformation following the creation of a critical defect. OP = Outer Periosteum, IP = Inner Periosteum.

Taken together, these results give new insights into critical bone defect regeneration in the calvaria and identify a few aspects of the modifications of the osteocyte network that are caused by the defect creation in the early stage of repair. The bone apposition on both sides of the calvaria, with a predominance on the IP surface, underlines the major role played by both periosteal components. Gene expression modulation in osteocytes highlights their responses to the defect creation, especially the spatial regulation of *Dmp1*, compared to the moderate time-dependent increase of *IL6* expression (Fig. 5). Whether these reactions contribute to the global repair process remains to be determined. Analyzing how periosteal structures and osteocytes could better support bone repair in a critical defect model might open new avenues for bone repair strategies.

## Materials and methods

### Biomaterial granules

Two different commercially available biomaterials from the company ZimVie (Westminster, CO, USA) were used. We chose granules with a diameter between 250 μm and 1 mm, either made of synthetic β−TriCalcium Phosphate (IngeniOs β−TCP) or made of natural bovine bone (CopiOs Xenograft).

### Scanning electron microscopy SEM

SEM imaging was performed on the raw biomaterials with a microscope TM4000 Plus from HITACHI (Tokyo, Japan). Samples were mounted on a carbon conducting adhesive placed on a sample holder. Gold (Au) sputter coating was performed with a Mini Sputter Coater Quorum SC7620 at a pressure of 4.10^− 2^ mbar in Argon. A constant current of 15 mA was applied for 1 min 30 s to allow the creation of a coating thickness of 13 ± 3 nm. SEM acquisition was performed at a pressure of 5 Pa, operating at 20 kV, detecting both backscattered electrons (BSE) and secondary electrons (SE).

### Inductively coupled plasma-atomic emission spectrometry ICP-AES

The biomaterial granules were immersed in ultrapure water and maintained in a sterile humidified incubator at 37 °C with 5% CO_2_ for 72 h. For each biomaterial type, 200 mg of granules and 2 mL of water were placed in 3 wells of a 6-well plate. The supernatants of the 3 wells per condition were collected and spun down to remove remaining particles. Each leachate solution was diluted to 10 mL with ultrapure water and analyzed by Inductively Coupled Plasma–Atomic Emission Spectrometry (ICP-AES). The concentrations of calcium (Ca) and phosphorus (P) released from the biomaterials were measured using a HORIBA Scientific Activa ICP spectrometer installed at Mines Saint-Étienne (France). The following emission lines were monitored: Ca 211.276 nm, Ca 317.933 nm, P 177.433 nm, P 178.221 nm, P 213.618 nm, and P 214.914 nm. Calibration curves were established using three standard solutions (1, 10, and 100 ppm) prepared for both calcium and phosphorus. Ultrapure water was used as a blank control. All analyses were performed in triplicate to assess analytical reproducibility. The limits of detection, calculated from the blank measurements, were 0.14 ppm for calcium and 0.06 ppm for phosphorus.

### Animals and calvaria defect model

Six-month-old adult male heterozygous Flk1-GFP mice were used for the entire study (36.1 ± 1.1 g)^42^. The lineage has been maintained by breeding with WT C57Bl6/J females for at least 10 generations in the animal facility at the University of Bordeaux (accreditation number A33-063–917), and the project received the authorization of the French Ministry of Higher Education, Research and Innovation (APAFIS#27986–2020110911537075 v5). All experiments were performed in accordance with relevant guidelines and regulations. The animals were housed together in cages in an enriched environment (houses and nesting) with a 12-hour day/night cycle and food (A03 breeding/A04 maintenance diets, SAFE, Augy, France), and water *ad libitum*. They were randomly assigned to the groups (Empty, Xeno and β-TCP, Day-3 and Day-14). A total of 48 animals were used in this study (*n* = 8 per group). Bilateral critical-size defects were performed in each mouse calvaria on the parietal bones as shown on supplementary figure S4. The head was shaved one day prior to surgery under anesthesia consisting of 4% isoflurane (2 L/min) via an induction chamber and maintained throughout procedure at 2% isoflurane (0.2 L/min) with a murine anesthesia nose mask. On the day of the surgery, the same anesthesia protocol was used. Additionally, the mice were injected intraperitoneally with an analgesic before surgery (buprenorphine 0.3 mg/mL, 0.1 mg/kg). An incision was made at the skull midline to expose the calvaria. The critical size defects were created using a trephine drill of 3.5 mm outside diameter (ACE Surgical Supply, Brockton, MA, USA). The defects were filled with one of the biomaterials described above or left empty. The three different conditions tested were: (1) defect left empty (“Empty”, *n* = 16), (2) defect filled with bovine bone particles (“Xeno”, *n* = 16); (3) defect filled β−Tri Calcium Phosphate particles (“β-TCP”, *n* = 16). The skin incision was closed with absorbable suture thread (4 − 0, Ethicon, Raritan, NJ, USA). The procedures were performed ensuring that the body temperature was maintained. The mice were checked every day after surgery. In the case of an identification of pain, buprenorphine 0.3 mg/mL, 0.1 mg/kg was injected intraperitoneally. They were euthanatized through cervical dislocation either 3 days post-surgery (*n* = 8 per condition), or 14 days after surgery (*n* = 8 per condition). The calvaria were harvested as described below.

### Sample preparation

The calvaria were harvested and cut in halves. The right side was snap frozen for laser microdissection as follows: it was foiled into aluminum and snap frozen using isopentane and liquid nitrogen, embedded in OCT and snap frozen again. Samples were stored at − 80 °C. The left side was fixed in 4% PFA (AntigenFix, Diapath, Martinengo, Italy) for 24 h and immersed in PBS 1X until microCT imaging. Once the microCT scans were performed, the samples of the six-month-old mice were decalcified in Microdec (D0053, Diapath, Martinengo, Italy) for 1 week. After decalcification, samples were immersed in sucrose 15% and 30% and embedded in OCT (SCEM Kawamoto C-EM001, SECTIONLAB Co. Ltd., Yokohama, Japan). Since the Microdec solution induced some DNA modifications, it prevented nucleus staining with DAPI on these tissue sections. Thirteen-month-old mice with the same defects, and whose calvaria were decalcified for 1 week in 10% EDTA in PBS, pH 7.4, were used for the sclerostin immunolabeling, phalloidin and DAPI staining.

### Microcomputed tomography (MicroCT)

#### Imaging

MicroCT scanning of bone samples was performed with a Skyscan 1276 high resolution, fast in vivo desktop device from BRUKER (Billerica, MA, USA), using an X-ray energy of 70 kV (200 µA), a voxel resolution of 12 μm, 3 frames average per rotation step of 0.4 °, exposure time 575 ms, on a 180 degree range of motion, with a 0.5 mm Aluminum filter. Images were reconstructed using the NRecon software (Brüker, version 1.7.4.6).

### Analysis

**Fig. 6:**
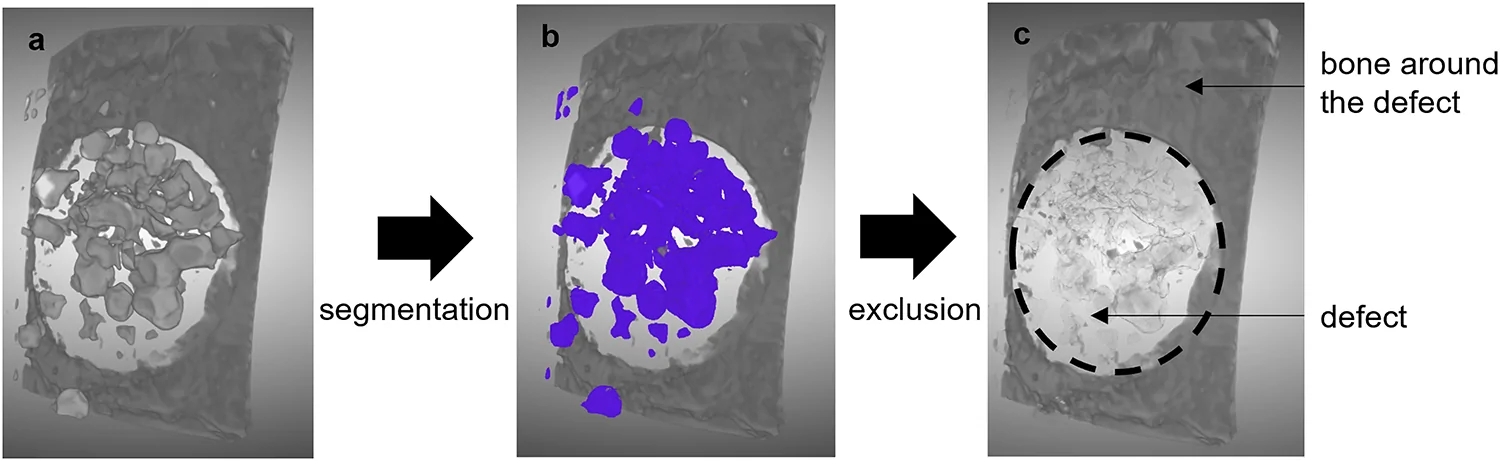
Method for MicroCT scan analysis using Dragonfly software. The Xeno granules and mouse bone inside and around the defect have close X-ray attenuations (**a**). To quantify the periosteal bone apposition around the defect, it was necessary to segment the biomaterial granules shown in purple (**b**) and exclude them from the defect. Thus, only the bone volume outside of the dashed circular line was quantified (**c**).

The software Dragonfly^43^(Comet Technologies Canada Inc., Montreal, Canada, version 2022) was used to perform the MicroCT images analysis. The aim was to evaluate the periosteal bone apposition around the defect. As bovine xenograft granules had the same radio opacity as mouse bone, we developed a method to specifically segment the bone substitutes and exclude them from the quantification (Fig. 6a). First, the images were reoriented. Then, they were cropped with the defect in the center, to analyze the same volume for all the conditions (34.1 mm^3^). For each image, inside the cropped volume, the biomaterial was segmented using the “point round brush” and the threshold upper Otsu (Fig. 6b). Further adjusted thresholds were set to fit the differences in radio-opacity of the Xeno and β−TCP materials. The pixel intensity values of the segmented volume were set to 0 to exclude the bone substitutes from the image (Fig. 6c), then we segmented and measured the volume of bone around the defect (outside of the circular dashed line traced in the Fig. 6c).

### Cryosections and histology

A MM cryostat (Brignais, France) was used for cryosectioning the decalcified tissue blocks. Blades specifically designed for hard tissue were used (MM35P, MM France). Sagittal sections with a thickness of 10 μm were performed using the Kawamoto method (cryofilm type 3 C 16UF, SECTIONLAB Co. Ltd.)^44^. All the steps were performed at −27 °C ± 1 °C. For each sample, one piece of tape with the section on it was fixed to a glass slide to perform Haematoxylin-Eosin (HE) staining. It allowed to stain the tissue in pink and the nuclei in dark blue. The glass slides were first immersed in tap water for 3 min to remove the OCT and in Mayer’s Hemalun (VWR, Radnor, PA, USA) for 2 min to stain the nuclei. After rinsing with tap water and ultra-pure water, the slides were immersed in a solution of Eosin 1% for 6 min (Eosine-225, RAL Diagnostics: 312730-0100) and rinsed again with tap water. It was followed by 7 dehydration steps with Ethanol 95% (2 min, x2), Ethanol 100% (2 min, x2), Ottix (2 min, x3). Finally, the slides were mounted with Diamount (Diapath) mounting medium. Sclerostin immunostaining, Phalloidin and DAPI staining were performed as described previously^45^. Sclerostin immunostaining was performed on sections from 6-month-old and 13-week-old mice, as follows: sections were rinsed for 5 min with PBS 1X, permeabilized with 0.1% Triton X-100 for 10 min and then rinsed with PBS-T (0.2% Tween 20 in PBS 1X). The sections were then blocked with PBS-T containing 1% BSA, and 0.3 M glycine for 1 h at room temperature, followed by incubation with the primary antibody (AF1582, R&D System, Bio-Techne France, Lille, France, 5µg/mL) in 1% BSA and PBS-T containing 3.3 µM phalloidin conjugated to Alexa-647, overnight at 4 °C. The sections were rinsed with PBS-T and then incubated with Alexa-488 tagged secondary antibody (2 µg/mL, A-11055, Invitrogen) for 1 h at room temperature. Finally, sections were immersed in DAPI (1 µg/mL, Thermo Fisher Scientific) for 5 min at room temperature to label the nuclei. Sections were rinsed and mounted with Fluoromount-G (Invitrogen). The same efficiency of sclerostin labeling was observed with the two kinds of decalcification, but nucleus staining with DAPI was efficient only after incubation of calvaria in the home-made 10% EDTA solution.

### Imaging and analysis

After HE staining, the sections were imaged for two different analyses. First, an Eclipse 80i light microscope (Nikon, Tokyo, Japan) equipped with a color camera (Nikon DS-RI2) at a 20x magnification to image the defect area. Two pictures per sample were taken, one on each side of the defect (nasal and occipital sides). For each picture, osteocyte nuclei inside the cortical bone were quantified manually using ImageJ/Fiji software^46^(1.54f; Java 1.8.0_172 [64-bit]) after having defined a surface of quantification (similar for all the groups). Secondly, a slide scanner (HAMAMATSU NanoZoomer, Hamamatsu city, Japan) was used to image the whole section for each sample at a magnification of 20x. The software NDP.view^47^(NanoZoomer Digital Pathology, HAMAMATSU Photonics K.K., version 2.6.13) was used to measure manually the thickness of the newly formed bone in different areas of the calvaria: outer periosteum (OP), inner periosteum (IP), close (within 600 μm from the defect edge), and distant (between 1.0 mm and 1.6 mm from the defect edge). Three measures per area for each sample were performed. Woven and native bone were identified based on their morphological characteristics in HE-stained sections. Woven bone appeared porous and was located on both sides of the calvaria. Native bone was observed centrally and despite being denser contained marrow cavities and blood vessels, with a clearly lamellar organization. In most cases, the boundary between woven and lamellar bone was distinguishable (Fig. 2a). Fluorescence imaging of sclerostin, Phalloidin and DAPI labeling was performed with a Leica TCS-SPE confocal microscope, using 20x, and 63x objectives and a zoom of 1.5.

### Laser-assisted microdissection

We collected osteocytes thanks to an optimized method of Laser Capture Microdissection previously described by our group^16^. Briefly, 5 μm-thick cryosections of non-decalcified calvaria were performed using the LMD Kawamoto film method (LMD film type 2 16, SECTIONLAB Co. Ltd.). Sections were microdissected using a 355 nm UV laser, coupled with an inverted microscope and software (P.A.L.M MicroBeam, Carl Zeiss, Jena, Germany). A 20x magnification was used and laser energy and focus settings were adapted according to the microdissected samples. Osteocytes inside the bone matrix were microdissected, and the bone pieces were catapulted into an adhesive cap (500 µL, Carl Zeiss) placed above the bone section. Between 1.3 and 1.4 mm^2^ were collected in a maximum time of 2 hours. For each sample, osteocytes were microdissected in two different areas, close (within 600 μm from the defect edge) and further (from 600 μm to the next suture) from the defect. Bone pieces were collected into a tube and stored in lysis reagent (MasterPure Complete DNA & RNA Purification Kit, Lucigen, Teddington, UK) at −80 °C until RNA extraction.

### RNA extraction and RT-qPCR

RNA was extracted following the recommendations from the MasterPure Complete DNA & RNA Purification Kit (Lucigen). Then, it was processed and analyzed according to an adaptation of published methods^48^. Briefly, cDNA was synthesized from total RNA by using qScript XLT cDNA SuperMix (Quanta Biosciences, Beverly, MA, USA). qPCR was performed with a LightCycler 480 Real-Time PCR System (Roche, Meylan, France). qPCR reactions were done in simplicate for each sample by using LightCycler 480 SYBR Green I Master (Roche) in a final volume of 10 µL. The qPCR data were exported and analyzed in the Gene Expression Analysis Software Environment developed at the Neurocentre Magendie (Bordeaux, France). Relative expression analysis was normalized against two reference genes tested as the most stable for the calvaria: *Ywhaz* and *Cyc1*. To determine the reference genes, samples from the “Empty” group at 72 h and 2 weeks were specifically microdissected far and close to the defect. RNA was processed as explained above. The Genorm method was used to determine the reference genes out of 9 candidates^48^. The relative level of gene expression was calculated with the comparative (2^−ΔΔCT^) method^49^. When analyzing the “Empty” condition, the calculation was done with the data obtained distant and 14 days after surgery as reference. When comparing the Empty, β-TCP and Xeno conditions, the data obtained in the “Empty” condition were taken as reference for the calculation. Primer sequences are reported in supplementary Table 1.

### Statistics

Statistical analyses were carried out using GraphPad Prism 10. We used either a Kruskal-Wallis test (KW) followed by Dunn’s multiple comparison test or a Two-way ANOVA test followed by a Tukey’s multiple comparison test. We considered *p* < 0.05 significant.

## Supplementary Information

Below is the link to the electronic supplementary material.


Supplementary Material 1


## Data Availability

“The data that support the findings of this study are available from the corresponding author upon reasonable request.”
