# Supplementary material for: Unexpected periosteal bone apposition including newly embedded osteocytes occurs around a mouse calvaria critical defect, independently of the presence of biomaterials
Source: Sci Rep. 2026 Jul 2;16:27097. doi: 10.1038/s41598-026-58356-7 (PMC13524924; doi:10.1038/s41598-026-58356-7)
Supplement: Supplementary file 1 [file 41598_2026_58356_MOESM1_ESM.docx]

**Supplementary material**

**Supplementary figure S1.** SEM images of bovine bone granules (CopiOs Xenograft, Xeno) and β-TriCalcium Phosphate granules (IngeniOs β-TCP) at 3 different magnifications (x25, x100, x500).

**Supplementary figure S2.** MicroCT images of the defect area and surrounding region at Day-3 and Day-14, in the Empty condition. The white arrows indicate the neoformed bone on Day-14.

**Supplementary figure S3.** Calculation of the distance around the defect in which the same volume of bone was produced after 14 days as would be required to completely fill the defect. The calculation is based on the approximation of a constant bone apposition thickness around the defect (60 μm).

**Supplementary figure S4.** Scheme of the dorsal view of a mouse calvaria after defect creation. Schematic representation based on X‑ray images.

**Supplementary Table1.** Primer sequences
